# A qualitative study into the perceptions and needs of fathers with a migration background on parenting regarding energy balance-related behaviors

**DOI:** 10.1186/s12889-025-21802-8

**Published:** 2025-02-11

**Authors:** Meredith L. Overman, Roel C. J. Hermans, Ibrahim Loukili, Mai J. M. Chinapaw, Abdellah Mehraz, Lauren Ekkelboom, Stef P. J. Kremers, Carry Renders

**Affiliations:** 1 Department of Health Promotion, NUTRIM Institute of Nutrition and Translational Research in Metabolism, Maastricht University, Maastricht, 6229 HA the Netherlands; 2 Amsterdam UMC location Vrije Universiteit Amsterdam, Public and Occupational Health, De Boelelaan 1117, Amsterdam, The Netherlands; 3 Amsterdam Public Health, Health Behaviors and Chronic Diseases, Methodology, Amsterdam, The Netherlands; 4 LeefstijlLab, Arnhem, 6814 BK Netherlands; 5 Trias Pedagogica, Wilhelminaplantsoen 1B, Diemen, 1111 CJ the Netherlands; 6 Department of Health Sciences, Faculty of Science, Vrije Universiteit Amsterdam, Amsterdam Public Health, Amsterdam, 1081 HV the Netherlands

**Keywords:** Fathers, Parenting practices, Multi-language research, Energy balance-related behavior, Healthy lifestyle, Cross cultural parenting research

## Abstract

**Background:**

Overweight among adolescents is worldwide still considered a serious public health problem. Although both parents influence children’s energy balance-related behavior, most studies have predominantly focused on mothers and white populations. Therefore, in this study, we contribute to the research by exploring the perceptions and needs of Dutch fathers with a migration background on parenting, specifically regarding promoting healthy energy balance-related behaviors among their children, and what motivates fathers to participate in parenting programs focused on these behaviors.

**Methods:**

We used a qualitative research design. Informal conversations (*n* = 2), semi-structured interviews (*n* = 11) and one focus group (*n* = 13) were conducted with professionals specialized in intercultural pedagogy and fathers participating in a parenting program organized by these professionals. Interviews and focus group were audio-recorded and transcribed. Atlas.ti 8 was used for theme detection, categorization, and classification using inductive and deductive approaches. The data was analyzed using grounded theory analysis.

**Results:**

Fathers joined parenting programs to improve their parenting skills and knowledge and address health and socio-cultural challenges. Furthermore, intergenerational differences were evident: second-generation fathers were more proactive in tackling parenting challenges related to healthy lifestyles. Fathers highlighted challenges related to parenting in two cultures. Although participating in the parenting program facilitated fathers in adopting a healthier lifestyle for both themselves and their families, improving communication with family members, and experiencing changes regarding gender dynamics within their household, influencing their teenage children, to adopt healthier habits remained a challenge, especially in comparison to younger children.

**Conclusions:**

A deeper understanding of the needs, perceptions, and experiences of migrant populations concerning parenting regarding the promotion of healthy energy balance-related behaviors among their children can lead to better-tailored health promotion programs that prioritize cultural and linguistic inclusivity.

## Background

Overweight and obesity among children and adolescents are considered a serious worldwide public health problem [1]. Globally, the prevalence has almost tripled from 4 to 18% between 1975 and 2016 [1]. In The Netherlands, 12.9% of children aged 4 to 17 have overweight, of which 3% have obesity [2]. Adolescence, in particular, is a vulnerable life phase when it comes to energy balance-related behaviors. Poor eating habits, insufficient physical activity, and sleep deprivation during adolescence contribute to weight gain and developing overweight [3]. In the long term, adolescents with overweight are more likely to develop obesity and associated health risks in adulthood [4]. If adolescents adopt healthy energy balance-related behaviors early, they are more likely to maintain healthy habits throughout their teenage and adult years [5]. As such, childhood and adolescence are crucial life phases for establishing lifelong healthy energy balance-related behaviors [5, 6].

Parents and other caregivers (in this article referred to collectively as “parents”) play a fundamental role in establishing and supporting their children’s healthy energy balance-related behaviors. They serve as providers, role models, and regulators of children’s behaviors [7–10]. Parents’ own eating and physical activity levels, for example, may directly influence their children’s eating and physical activity behaviors– often referred to as parental modeling [11, 12]. Moreover, parents adopt parenting styles that set the overall emotional climate within the family and use parenting practices that shape the energy balance-related behaviors of their offspring. Previous research found that some parenting practices such as creating a healthy food home environment, employing positive parental communication strategies and limiting exposure to unhealthy foods are associated with healthy energy balance-related behavior [10, 13–16]. Hence, targeting parents is crucial in interventions focused on energy balanced-related behavior to prevent and treat overweight among children and adolescents [13, 17].

While the role of the family environment in children’s energy balance-related behavior has been studied extensively, traditionally mothers tend to participate more often in interventions targeting parents [18–27]. However, parenting practices of mothers and fathers may affect children’s energy balance-related behavior differently [12, 16, 23]. Although the role of fathers has recently gained more attention in research regarding energy balance-related behavior in children and adolescents [15, 25, 28–30], current knowledge regarding paternal influences is still limited.

An additional factor influencing children’s energy balance-related behavior is the cultural background of parents and children. Many parenting practices are shaped by cultural norms and values [31–33]. Previous research suggests that there are cultural differences in parental values, goals, and practices in general, and highlights that parenting practices need to be understood within their cultural context [31–36]. However, most studies on parenting fail to adequately represent parents from diverse cultural backgrounds, particularly fathers with a migration background [27]. For example, the majority of the existing research on dietary patterns focuses on a predominantly white populations [7, 13]. This lack of representation is part of a broader historical trend in which minority communities have been underrepresented in health-related research. The lack of diversity in health-related research studies contributes to inequities, ethical concerns, and reduces scientific rigor in health-related research. When evidence-based interventions are based on selective evidence, these interventions are less likely to connect well with families from communities currently underrepresented in research. Therefore, in this study, we aimed to address these research gaps by exploring the perceptions and needs of fathers with a migration background on parenting, particularly regarding promoting healthy energy balance-related behaviors among their children. Specifically, we aimed to understand their motivations for participating in a parenting program and to identify factors that could enhance the alignment of such programs with their needs. Using a parenting program focused on energy balance-related behavior that targets fathers with a migration background as a case study, this research sheds light on the potential benefits of such interventions. By prioritizing the voices of fathers with a migration background - particularly those of Turkish and Moroccan origin, as these groups represent one of the largest migrant groups in the Netherlands and are known to face an elevated risk of being overweight from an early age - this study provides valuable insights into culturally sensitive strategies for engaging fathers in parenting programs [37, 38].

## Method

### Research context

This study is part of the Lifestyle Innovations based on youths’ Knowledge and Experience (LIKE) program and is carried out in collaboration with Trias Pedagogica [39, 40]. LIKE is part of the Amsterdam Healthy Weight Program (AHWP). AHWP is a local government-led whole systems approach aiming to reduce childhood overweight in Amsterdam, the Netherlands, before 2033 [41]. LIKE combines a systems dynamics and participatory research approach to develop, implement, and evaluate action programs that contribute to reducing the complex problem of childhood overweight among children and adolescents in three lower socioeconomic districts in Amsterdam East [42].

### Study design

Given the exploratory and interpretive nature of the study, we chose a qualitative research design [43, 44], executed in the context of the healthy lifestyle track of the Parenting Debates of Trias Pedagogica (from here on referred to as parenting program) that was implemented between 2019 and 2020. We conducted interviews and informal conversations with fathers, debate leaders, and key figures who work with fathers in lower socioeconomic districts in Amsterdam to gain a better understanding of the perceptions and needs of fathers for interventions on parenting regarding energy balance-related behavior. Subsequently, we conducted a focus group with a different group of fathers to verify the interview findings and collect richer data on specific topics [44]. To better understand the philosophy and content of the healthy lifestyle track, we held preliminary interviews with the developers of the parenting program.

All participants received a letter informing them about the study’s aims and method and provided written or audio-recorded informed consent. Subsequently, we read the informed consent form aloud in Dutch or translated it to Moroccan Arabic. To ensure the participants’ privacy, researchers coded participants’ names (e.g., R1, R2) and removed personal characteristics. The principal researcher (MO) and a researcher in training (IL) scheduled all informal conversations and interviews at the participant’s convenience. Participants received a €50 gift card by mail or in person after participation. The Medical Ethical Committee of the VU University Medical Centre (2018.234) approved the research protocol. Data was securely stored on the internal drive of the Amsterdam UMC and archived for five years after the completion of the study.

### Parenting program as a case study

Trias Pedagogica– a unique consultancy and social organization specialized in parenting skills and practices, intercultural pedagogy, and fatherhood - developed a program in 2012 called “Parenting Debates”. Trias Pedagogica was established from a personal need of its founder. As a child, despite having his father around, the founder felt the absence of the guidance and support of his father. Furthermore, he observed that most parenting programs were primarily focused on mothers. His personal experience and the underexposed role of fathers in pre-existing parenting programs led to his passion for exploring the role of fathers in their families and the development of Trias Pedagogica. With the parenting program, Trias Pedagogica strives to empower the father’s role in the family [39, 45]. The main goal of the parenting program is to reach and support fathers who are not attracted to mainstream services [39]. Fathers can participate for free in the parenting program, which is held during the evenings and weekends. Facilitated by a debate leader, fathers cover different parenting topics through different work forms, such as a board game, card game, statements, and examples in the form of a video, animation, or case study. Fathers receive tools and information to improve their knowledge, skills, and parenting practices [39, 45]. The debate leaders facilitate the parenting program for both (grand) fathers, (grand) mothers, and other caregivers with a migration background living Amsterdam. All debate leaders have a migration background and speak the same language as the parents participating in the debate, which helps them effectively facilitate debates for parents from diverse backgrounds. Additionally, they often share the same cultural background as the parents.

To better understand the context of the study, we provide a brief description of the parenting program that was implemented between 2019 and 2020. The parenting debates consist of eight meetings divided into three modules: the general parenting debates, the in-depth debates, and a return day. The first module covers four themes: fathers’ experiences, fatherhood, parenting alignment, and balancing parenting [39]. After that, fathers can choose from seven topics: prevention of radicalization [46], prevention of domestic violence [47], prevention of youth criminality [46], promotion of sexual resilience and three modules that focus on health and lifestyle: the first 1000 days, healthy lifestyle and promotion of mental health. This study focuses on the ‘healthy lifestyle’ module, wherein the main topics are diet, physical activity, and sleep. A few weeks after the in-depth track, a return day occurs where fathers review their experiences concerning their behavior. Trias Pedagogica facilitates over fifty groups each year, with an average of 16 parents per group. Through the general parenting debates they reach approximately thousand parents per year, of which 70% fathers. With regard to the healthy lifestyle track, they facilitate 7 to 8 groups of fathers per year. To conclude, fathers are involved in the evaluation of the parenting program.

### Recruitment

In order to recruit participants with different roles in the parenting program, convenience sampling, and a snowball sampling method were used [44]. The principal researcher (MO) established a relationship with the developers of the debates prior to the current study. MO attended several sessions of the parenting program to gain a better understanding of the program. Furthermore, MO had several informal conversations with Trias Pedagogica to get to know each other better and discuss topics related to parenting and healthy lifestyle. Trias Pedagogica provided the research team with a selective sample of debate leaders and key figures who were willing to participate in this study. Trias Pedagogica selected the key figures within this study:fathers with an extensive network in Amsterdam (East) and who followed the Healthy Lifestyle track in Amsterdam East in 2020. These key figures had a dual role as gatekeepers within the community and as a father. We asked the debate leaders and key figures to assist in recruiting fathers who attended the Healthy Lifestyle track through their social networks. The principal researcher (MO) or researcher in training (IL) contacted nineteen potential participants by telephone. Thirteen participants agreed to participate in an individual interview or informal conversation: seven fathers, two debate leaders, two key figures, and two methodology developers. Thirteen fathers participated in a focus group interview of whom two, a father and a key figure, also participated in an individual interview.

### Setting and data collection

Data collection took place between March 2021 and May 2021, during the Islamic month of Ramadan. As the Dutch COVID-19 measures at the time prohibited in person gathering, the informal conversations and interviews were held online using video conferencing software ZOOM (*n* = 6), Microsoft Teams (*n* = 2), or through a phone call (*n* = 5), depending on the participants’ preferences. In May 2021, some COVID-19 measurements were released, which allowed us to conduct one focus group in person with an existing group of (grand) fathers who participated in the Healthy Lifestyle track in 2019. The principal researcher (MO) and a researcher in training (IL) conducted five interviews and two informal conversations together, and IL conducted four individual interviews and the focus group interview. The informal conversations and interviews took approximately 30 to 83 min, and the focus group took roughly 60 min.

Our research team consisted of cis-gender women and cis-men; with a Dutch, Dutch Chinese-Surinamese, Dutch Afro-Surinamese, and a Dutch Moroccan background. In addition, several of us related to the experience of partners and children and have a Christian, Catholic or Islamic upbringing or spiritual affiliations with Buddhism. Our scholarly backgrounds covered medical and cultural anthropology, public health, human movement science, health sciences, social and behavioral science, pedagogy, and epidemiology. Although certainly not exhaustive, the diversity within our perspectives let to fruitful discussions of our data. With this study, we hope to contribute to more diverse and inclusive research by including diverse experiences, perspectives, and representations in research and within the research team, thus making science more AWESOME; All-inclusive, Worldwide ranging, Equitable, Sincere, Open-minded, Mindful of our own implicit bias, and Essential [48].

The principal researcher (MO) developed a semi-instructed interview guide for the informal conversations, interviews, and focus group. The interview guide included subjects related to participants’ motivation, expectations of the sessions, experiences, and needs concerning the debates and learned lessons. The interviews and focus group were audio recorded and stored securely. After each interview and the focus group, at least two members of the research team reflected and took notes on the content and process of the interviews and focus group. During the informal conversations, we made field notes. During the focus group, we made observations and took notes. During the interviews, we asked each participant the following information: gender, age, country of birth, cultural, migration and religious background, and other details such as type of employment, number and age of children, and what year they participated in the parenting program. We asked the participants in the focus group for this information after the session. We conducted the interviews, informal conversations, and the focus group in the participants’ preferred language, which included Dutch (*n* = 6), Moroccan-Arabic (*n* = 4), a combination of Dutch and Moroccan-Arabic (*n* = 1), or Turkish (*n* = 1). IL conducted four interviews and the focus group in Moroccan-Arabic. MO and IL, assisted by a translator (one of the key figures), conducted one informal conversation in Turkish.

### Language, translation, and qualitative data analysis

IL simultaneously transcribed verbatim and anonymized the audio-record interviews and focus group. As not all researchers were familiar with the Arabic language, IL transcribed the files recorded in Arabic directly into Dutch– at data collection- and we conducted the qualitative analysis on the Dutch transcripts [49]. One researcher (IL) translated five interviews and the focus group from Moroccan-Arabic to Dutch. To reduce interpreter bias, a bilingual colleague, proficient in both Moroccan-Arabic and Dutch language, checked six transcripts for accuracy [49, 50]. MO and IL translated each theme and quote from Dutch into English, meaning that meanings and nuances in participants’ stories might have gotten lost in the translation process [50].

We conducted a grounded theory analysis to comprehensively understand the participants’ perspectives and experiences regarding the parenting program [44]. We utilized the qualitative data analysis software ATLAS.ti (8th edition) to inductively code and analyze and identify recurring themes, analyzed structures, meaning, and context within the transcripts of the interviews, focus group, field notes of the informal conversations, reflection notes, and observation notes. To ensure the accuracy and reliability of data interpretations, two researchers (MO and IL) independently open-coded the informal conversations, interviews and focus group and compared codes until reaching a consensus. After open coding, axial coding took place by creating codes that reflect multiple fragments, and MO and IL grouped codes in themes into a preliminary code tree. We deductively determined the main categories of the code tree and initial codes from the interview guide and data. MO, IL, CR, and RH regularly discussed the code tree and themes and compared the codes to find relevant connections. This resulted in the key elements that reflected the participants’ various experiences: selective coding was applied [44]. The researchers discussed all differences until reaching a consensus.

## Results

### Participants

In total, 24 participants agreed to participate in the study, of whom eighteen fathers, two debate leaders, two key figures, and two methodology developers of the parenting program. The fathers resided in different districts in Amsterdam East. The age of the participants ranged from 27 to 76 years, and some of the fathers interviewed were also grandfathers. In addition to the methodology developer and the debate leaders of Trias Pedagogica, the participants were either employed (*n* = 7) in various sectors, including education, sports, community foundations, municipality, and public transportation, retired (*n* = 5), unemployed (*n* = 5) or were receiving social benefit (*n* = 3). Three participants had two employments. Table 1 presents the key characteristics of the participants.

**Table 1:** Descriptive of the participating fathers, key figures, debate leaders, and developers of the parenting program

Gender (*n*)	*n* = 24
Female, *n* (%)	1
Male, *n* (%)	23
**Age in years**,** mean (range)**	57 (range 27–76)
**Country of Birth (** *n* **)**	
Morocco	19
Turkey	2
The Netherlands	3
**Religion (** *n* **)**	
Islam	23
Atheist	1

Table 2 presents the four main themes we identified: (i) motivation to participate in parenting programs, (ii) intergenerational differences, (iii) the family environment, and (iv) gender-specific expectations of parenting. Through the four main themes, we identified three common contextual considerations that were important for fathers in parenting programs: (1) the program should be in the preferred language of the participants, (2) the program should take into consideration the language proficiency of the participants, and (3) a program should have a good ambiance, where mutual respect is a central point in the interaction during a parenting program.

**Table 2:** Main perceptions and needs of parenting programs and parenting related to energy balance-related behavior

	Main themes	Sub-theme	Contextual considerations for parenting programs
1	Motivation to participate in parenting programs	a) Acquiring and share knowledge	a) Programmed in preferred language b) Language proficiency c) Good ambiance and mutual respect
2	Intergenerational differences	a) Parenting b) Level of motivation	
3	The family environment		
4	Gender-specific expectations of parenting		

### Theme 1 - Motivation to participate in parenting programs

Fathers identified several reasons to participate in a parenting program focused on energy balance-related behavior. Most fathers mentioned that their motivation was to gain more knowledge and new ideas about parenting, nutrition, and sports and share this knowledge with family and friends.

“I find this very important for me and for my family and those I know, so family, acquaintance and friends. This so I can help them [and] share ideas with them that might be helpful in terms of eating or exercise.” (R8).

In addition, some fathers joined the parenting program because they wanted tools to manage their own health conditions (e.g., diabetes). Furthermore, fathers mentioned that they wanted to enhance their communication skills when discussing energy balance-related behaviors with their partners and teenage children. They also wanted to share and discuss their experiences and perspectives and learn from the experiences and expertise of other fathers. This is because they considered healthy lifestyle an important topic.

[My] motivation was R5 [one of the key figures], he motivates us. [The parenting debate] was a good initiative and was quite nice to participate. Also [to have] other insights at home, how to deal with [parenting and healthy lifestyle] at home. Everyone has their own way of doing things, you learn a bit from each other and from the person [the debate leader], who provides us with information.

(Father, focus group)

Besides the need to become in contact with like-minded fathers, the results also demonstrated that fathers encourage each other to attend the parenting program.

“Here we are like brothers; we are neighbors and live next to each other. If I don’t go today, I get 5 to 6 calls asking where I am and whether I am sick or something. If someone doesn’t come, we look for him.” (Father, focus group).

Furthermore, the key figures emphasized the importance of repetition and having enough sessions in parenting programs. The fathers confirmed this need. Moreover, fathers mentioned that in addition to discussing parenting and energy balance-related behavior topics, they wanted to do things together. For example, fathers proposed parenting programs with group activities centered on physical activity. According to them, these activities would encourage fathers to increase their physical activity by participating in sports or walking frequently with a group in their neighborhood. One of the fathers believed that by raising awareness and expressing dissatisfaction about the current obesogenic environment, fathers could contribute to a healthier environment not only for themselves and their families, but also for other residents of Amsterdam. Furthermore, he expressed his hope that together they could encourage the municipality to reduce the availability of unhealthy food in their city. In addition to their intrinsic motivation, fathers also indicated that they were encouraged to participate in the parenting program by the key figures.

In addition to parenting programs focusing on energy balance-related behaviors, fathers were also interested in discussing other topics related to parenting, such as aging, challenges related to migration and integration, institutional racism within education, and behavioral norms and values. One of the fathers stated:

“We shouldn’t just talk about food.” (Father, focus group).

A few fathers, for example, wanted to learn more about parenting in general since they were worried about their children’s future. They mentioned that many Moroccan youth in the Netherlands have a bad reputation, and the media often only covered topics about adverse behavior, e.g., stealing, vandalism, and drug abuse. Fathers struggled with the notion of being Dutch and the different consequences that came with being Dutch citizens with a migration background. This indicated that fathers had many other concerns they wished to address beyond topics related to a healthy lifestyle. Fathers and key figures emphasized that many existing programs failed to reach them because they focused on just one topic. One participant stated that other organizations preferred to focus on simple interventions such as cooking and having fun and thereby avoid more important issues parents have to deal with.

“Because the other organizations here do things that are easy, they want less headaches. Therefore, what they want to do is very easy. (…) They want to organize activities that they consider fun, some cooking, sewing activities, small talk, but the real issues, such as financial problems and stress, are ignored by these organizations.” (R7, key figure, Focus group).

Many fathers struggled with multiple health issues such as diabetes and depression, but also with other problems. The fathers mentioned that the complexities of personal problems and the various needs with regard to these problems were separate from the available programs.

Fathers expressed that current programs tackling parenting in relation to energy balance-related behavior should also include other topics of concern to them and their families. One of the developers of the parenting program also mentioned that other parenting programs struggled with reaching fathers with a migration background.

“It is actually crazy that [the parenting program is] necessary, that other parenting programs do not succeed in reaching this target group.’’ (R1).

Furthermore, debate leaders said that the themes and propositions within Trias Pedagogica’s parenting program aligned with the fathers’ needs. According to the debate leaders, this was due to the regular evaluation of the parenting program by the developers, debate leaders, and fathers. Due to this, fathers have been involved in developing and improving the parenting program.

According to the fathers, those with a first-generation migration background– often grandfathers- sometimes find it challenging to read and write in Dutch, while those with a second-generation migration background have fewer language difficulties. These language differences could complicate engaging in a parenting program for some fathers. Furthermore, many fathers stated that speaking Moroccan-Arabic was essential for their motivation to participate in a parenting program as they can better express themselves, engage in discussions, and share their thoughts. The possibility of speaking in their native language was also an important factor in participating in this study.

“Because as [researcher IL] speaks Arabic to us, you have gained experience and knowledge from your parents and family, so you know what we are going through and experiencing. This makes discussing things easier. A [white] Dutch person who comes, who comes to try and help you, has no knowledge of the situation.” (Father, focus group).

Conducting the interviews and focus groups in the preferred language, and a shared cultural understanding between researcher and participants lowered the barrier to participate. Fathers also mentioned that spirited debate and mutual respect led to good ambiance, and a sense of bonding, which in turn encouraged fathers to actively participate in parenting programs.

“The ambiance, how it is handled, the respect between the participants and [the debate leaders] who give the meetings. People listened well to each other and there were good discussions among the participants and between the participants and the teacher.” (R24).

To summarize, the motivation of fathers with a migration background to participate in a parenting program is strongly related to culturally responsive facilitation, inclusive language practices and the cultivation of mutual respect and peer solidarity.

### Theme 2 - Intergenerational differences

Regarding specific parental needs and preferences, some fathers experienced no intergenerational differences when it concerns parenting. One father explained that all parental concerns are similar.

“(…) the question is always how our children can be the best when it comes to education.” (Father, focus group).

Another father added that if there were intergenerational differences, a grandfather with more experience could share his experiences with his son. However, other fathers experienced differences in level of motivation and participation between fathers and grandfathers. According to one of the participants, the grandfathers just came to attend and spend time. They were less motivated and had fewer questions about parenting related to energy balanced-related behavior than second-generation fathers. Second-generation fathers came to discuss and deal with the challenges they faced. One of the fathers explained the following:

“(…) the second-generation, (…) we have our children here. We have to ask more questions than the rest. Because we have children, we experience problems related to sports, food and eating. [First-generation fathers] are past that.” (R10).

According to the fathers and the debate leaders, first-generation fathers, who have adult children, engaged less during the parenting program because they were past the phase of parenting. However, some fathers acknowledged that some first-generation fathers used the knowledge gained from the parenting program to improve the lifestyle of themselves, their children, and their grandchildren.

When it concerned raising children, many fathers mentioned that they were very much aware that they raised their children in a different country than their country of birth, with different habits, living environments, and conditions. Some fathers stated that some first-generation fathers still saw themselves as guests in this country, while others saw themselves as a part of the Dutch community. The debate leaders also mentioned that parenting in two cultures was a recurring topic. One of the fathers disclosed how he explained to his children what it meant to be a child with a migration background in the Netherlands and how to behave accordingly.

“There are black people, Turkish, Pakistanis, we have a whole world in this Netherlands, and everyone has their own culture. And you my child should treat them well and respect them. And you should say good morning so that others feel safe, because we are guests in this country. And you have to explain things to [your child] about this country and that we are from Morocco and that these people treated us well and gave your grandfather and me a house.’’ (Father, focus group).

Conversely, one of the debate leaders emphasized the importance of maintaining your own identity when it concerns parenting.

“We talk about parenting in two cultures, which means integration is important. Integration is also a word that is very loaded, because people (…) confuse integration and assimilation. The media too, the media talks about integration but they mean assimilation, you really just have to become Dutch. I will never be accepted as Piet, never, so then I will stay R4, myself (laughter). But [there must be] something from the other culture that suits me. But there are [fathers] who think if you’re going to tell me that, (…) then I may be putting a piece of my identity aside. And I always say no, you don’t have to.” (R4).

By stating this, the debate leader embraces bicultural parenting as a positive childrearing approach that allows families to maintain their identity alongside the country’s culture in which parents settle.

### Theme 3 - The family environment

Most fathers mentioned that attending the parenting program made them and their families adapt to a different lifestyle. One of the fathers stated:

“We have adapted many things, our diet, our lifestyle. We have changed a lot of things. For example, moving or walking. (…) I do sports activities with my family, and I also try to eat healthy [by eating] fruits and vegetables and [by not] eating after 8 o’clock in the evening and [by] drinking more water.” (R8).

Furthermore, fathers indicated encouraging their children to spend less time using a computer or a phone. Moreover, they stated that the parenting program made them spend more time with their children and do more activities together, such as grocery shopping or cycling. Some fathers also mentioned that the parenting program improved their relationship with their partners. They became more involved in the household activities such as cleaning the dishes and doing groceries with their families. The debate leaders also shared these observations during the interviews. Fathers indicated that they actively incorporated insights into their daily lives by communicating them with their partners at home. One of the fathers adds that:

“ (…) parenting is something that changes with the development of humans and technology. It does not remain stable.” (R11).

This idea might help parents to encourage their children to obtain healthy habits. Conversely, a few fathers said that they could not influence their children’s lifestyle. They mentioned that their children were too old to be influenced and could still buy unhealthy food when they go outside.

“I learned a lot [at the parenting program], but if you explain it to the kids who are big, over 18 and one who is 17 [years old]. But they are big, it’s hard to make them obey you and listen and see the benefits. They have control over themselves.” (R10).

A father stated that the influences outside their homes, the age of their children, and the growing independence that comes with age, made it difficult for fathers to influence their teenage children in adopting and maintaining healthy choices. One of the fathers discussed how the presence of unhealthy food in their homes made it challenging to avoid eating it themselves and to set rules for their children. Another father explained that, due to his illness, he has returned to eating foods he consumed while living in Morocco, transitioning from processed food to consuming more olive oil, lentils, and fish. He elaborated that this dietary transition helped him to manage his illness. This example demonstrates that dietary practices from one’s country of origin can be instrumental in managing chronic illnesses like diabetes, fostering both physical well-being and a meaningful connection to cultural heritage. However, he believed he could not persuade his children to follow the same eating habits. He admitted that, with this transition, he did not have meals with his children anymore. He stated:

“Not only families or parents, but also the country and the municipality or those who govern and the companies [involved in the food system] should reduce this poison, unhealthy food.” (R10).

This father advocated for reducing the cost of healthy foods while increasing the price of unhealthy foods.

### Theme 4 - Gender-specific expectations of parenting

The different parenting expectations of mothers and fathers were frequently mentioned. Most fathers explained that they had changed their perspectives regarding the responsibilities of fathers and mothers within the household and with regard to parenting.

Father #: “Yes certainly, when one goes home now, for example during Ramadan, [you arrive home] half an hour beforehand to help in the kitchen. And after dinner he [the father] helps, and certainly the children see this, and they learn from this to do it later with their own wives.” (Focus group).

Father #: “You also convey a message to your children. We, Moroccan [men] honestly don’t do much at home, not how it should be actually. While the Prophet, peace be upon him, helped his partner. So, who are we not to help. For the woman it is also hard. When you help the woman then she also sees it and is encouraged.” (Focus group).

A Turkish father shared that his partner appreciated his involvement in parenting programs. He used to believe that caring for his children’s sleeping and eating habits was solely a mother’s responsibility. Since participating in the parenting program, he also encouraged their children to go to bed on time and eat healthy. This effort resulted in better alignment of both parents regarding parenting. In addition, he proudly added that he started to spend more time with his grandson and cycled 30 to 45 min every day with him since he participated in the parenting program.

The COVID pandemic that started in 2020 also highlighted gender differences within the households. Families faced various difficulties with school closures, children staying home, and parents working remotely or losing their jobs. One of the fathers explained that fathers who were less used to spending a lot of time with their wives and children encountered problems within their families. He elaborated that before COVID, fathers often arrived home late from work, and when the children went to school early, they were still sleeping, resulting in less time to interact and parent.

## Discussion

This study aimed to explore the perceptions and needs of Dutch fathers with a migration background on parenting, specifically regarding promoting healthy energy balance-related behaviors among their children. Fathers were motivated to join a parenting program when it enhances knowledge about health and parenting, improve communication skills, and address broader issues like migration challenges and concerns about their children’s futures. Important features of a parenting program were a comprehensive approach, acknowledging multifaceted problems and prioritizing cultural and linguistic inclusivity. Fathers had different motivations to participate (e.g. desire to learn more) and identified a mixture of preconditions that parenting programs should meet (e.g. inclusive language, good atmosphere). Our findings emphasize the importance of creating a good atmosphere, which involves culturally responsive facilitation, inclusive language practices, and fostering mutual respect and peer solidarity in encouraging fathers with a migration background to participate in parenting programs. These elements should be considered key components in the design and implementation of future parenting programs aimed at fathers with a migration background.

Most fathers reported that the parenting program led them and their family to adopt healthier lifestyles and spend more time with their (grand) children– in general, but more specifically related to eating and exercising together. Recent research has shown the crucial role of fathers in shaping their children’s energy balance-related behavior. Acknowledging and addressing the influence of fathers can lead to more effective interventions and better health outcomes for children [16, 22, 51–54]. Furthermore, there is an association between the weight status of fathers and their children. This association is mediated by the physical activity levels of both fathers and children [55]. Therefore, it can be concluded that engaging in physical activities together as a family can be a promising intervention strategy [56]. While the role of fathers in children’s energy balance-related behavior is important, some fathers expressed challenges in influencing their older children’s habits due to external influences, their growing independence, and differing dietary preferences. The growing independence of their children makes it more challenging for parents to set, monitor, and enforce rules regarding healthy behaviors. Furthermore, fathers increasingly recognized and adapted their roles in parenting and household responsibilities, with many noting positive changes after participating in parenting programs. The COVID-19 pandemic further underscored gender dynamics in households, emphasizing the discrepancy in time fathers traditionally spend with their families.

Previous research suggests cultural differences in parental values, goals, and practices exist, and highlights that parenting practices need to be understood both between cultures and within their cultural context [31]. In addition, immigration is a complex life transition that can bring about significant changes in familial roles, responsibilities, and structures [27]. The family structure and the father’s traditional role when immigrating are often challenged, as their authority declines when parents become reliant on their children as intermediaries in the host country, primarily due to language barriers [27]. Our results showed that fathers noticed varied experiences regarding intergenerational differences in parenting, with second-generation fathers being more engaged in addressing challenges, while some first-generation fathers attended the parenting program more passively. In contrast, a study of Turkish fathers with a migration background in Germany found that first-generation fathers were more involved in their children’s development then second-generation fathers [57, 58]. In addition, our study underlined the challenges that come with raising children with dual cultural identities in a new country. It is often assumed that integration becomes easier with each subsequent migratory generation. However, second-generation fathers, despite not having migrated themselves, face unique challenges as they navigate two different cultures, which has consequences for their adaptation and parenting behaviors [59]. Migrant parents are confronted with both the normative challenges of parenting and have to adapt to the norms, values, and expectations of a new country [57]. This dynamic not only shapes parents’ parenting styles but also influences the formation of their children’s cultural identities. Hence, navigating parenthood between two cultures becomes challenging due to the complex interplay between preserving the cultural heritage and facilitating assimilation into the host society [57, 60].

### Conditions for parenting programs

When developing father-focused parenting programs targeting energy balance-related behaviors within the home environment, the present study identified several conditions that parenting programs should meet. First, it is crucial the parenting program aligns with the participant’s needs and their cultural and social backgrounds. Broadening the focus on healthy living to address underlying problems and multifaceted family issues can enhance, trust and understanding among participants [61]. Additionally, it is important to have facilitators who possess appropriate skills, training, and a deeper understanding of the cultural and social background of fathers, with particular attention given to language proficiency and language skills [61].

The participants in our study stated that a parenting program should not be a one-time event, emphasizing the need for multiple sessions. In addition, parenting programs should be designed to be neither childish nor pedantic but instead aim for a balance in content. While knowledge sharing is important, it alone does not create healthy energy balance-related behaviors within the home environment. Malek and colleagues [61] highlight that parents prioritize lifestyle interventions that involve and facilitate the family and key people around them. Although the fathers did not mention their family’ s involvement in the parenting program, they noted that attending the parenting program led to positive changes in communication with their partners and also improved their relationship.

The development of parenting programs also benefits from considering broader societal debates, such as intensive parenting cultures. This approach requires parents to invest significant time, money, and energy in the belief that it will lead to better outcomes for their children [60, 62, 63]. A key critique of intensive parenting lies in its assumption that the family environment is seen as the single strongest influence on children’s development, including their health and wellbeing. This perspective places excessive pressure on parents while overlooking broader social, economic, and environmental factors that influence children’s development: factors often beyond parents’ control. While there is clear evidence linking parental childrearing styles to improved physical health outcomes in children, there is limited research on how parenting styles impact children’s psychological well-being. The existing research suggests that intensive parenting may negatively affect children’s psychological well-being, particularly as they grow older [62]. Moreover, intensive parenting establishes normative standards that can leave parents, especially those from socio-economically disadvantaged backgrounds, feeling judged or inadequate if they cannot meet these monocultural standards [60, 62, 63]. This emphasizes the need for adopting a more comprehensive approach to parenting programs, considering the social, economic, and environmental factors influencing children’s energy balance-related behavior. Furthermore, involving the target group in the development and evaluation of parenting programs contributes to better tailored parenting programs. Notably, in this study, fathers’ engagement in the parenting program and its evaluations had contributed to shaping and enhancing the parenting program, making it genuinely tailored to their requirements. For example, based on the father’s needs, the parenting program module focusing on health and lifestyle has been redeveloped into a combined module of eight meetings focusing on positive health, diet, physical activity, sleep, a limited budget, and mental health.

### Contextual considerations for parenting programs

Parents with a migration background are often perceived as a group who is “hard to reach” [64]. Based on our study, we can conclude that current interventions do not sufficiently strike the right chord (i.e. too little attention to their specific needs, discussing difficult topics is avoided) and there is often insufficient attention to creating a context in which people feel comfortable (e.g. language proficiency, use of multiple languages as well as fostering mutual respect). This lack of attention to these factors leads to intersectional inequalities in families’ access to services. In line with Boag-Munroe and Evangelou [64], we should change the narrative and challenge labeling groups as “hard to reach” or “hard to engage”. Instead of assuming that certain groups are hard to reach, we should focus on aiming to understand what can be done to make services accessible and relevant to parents. Thus, it is not that the parents are hard to reach, but that the services are not adequate for their needs. Underlining this paradigm shift, Trias Pedagogica, a service that is experienced in making itself relevant and accessible to parents with a migration background, engages thousands of fathers in their programs on a yearly basis.

Furthermore, this study highlights the benefits of adopting a bicultural approach in health promotion programs, emphasizing the importance of integrating different cultures and culturally specific practices. Such an approach facilitates mutual learning and encourages healthy behaviors by combining beneficial habits from various cultural contexts. It also challenges the idea that the most effective health promotion methods come solely from host countries and that local experts unidirectionally impart these methods. Instead, the findings indicate that many health-related concerns expressed by migrants are influenced more by their living conditions in the host country than by factors related to their country of origin. This perspective advocates for a more inclusive and reciprocal model of health promotion that considers the lived experiences and cultural assets of migrants.

### Strengths and limitations

Informed consent forms were not translated into Moroccan-Arabic or Turkish, this might have influenced the inclusivity within the research. Furthermore, a considerable proportion of the participants had a Moroccan migration background, and therefore, the results of this study may not be representative of the population of fathers with a migration background in Amsterdam. Moreover, while we emphasize the importance of migration background, some of the results may also be influenced by socioeconomic factors since our sample included fathers with a migration background and limited financial resources [65]. Therefore, we were unable to isolate or study these factors independently. Furthermore, this study included a sample of fathers who were already engaged in a parenting program. The fathers who did or could not participate or were not approached may have different needs and perceptions from those who were included. Despite the limited generalizability of the results, the findings do contribute to the inclusion of perspectives and needs of fathers with a migration background regarding parenting programs focused on energy balance-related behaviors. We reached data saturation with our sample size as no new themes emerged after the final interviews and focus group. This aligns with established guidelines indicating that saturation is often achievable within similar sample ranges, particularly in studies involving homogeneous populations and clearly defined research objectives [66].

As researchers actively engaged in this study, we were able to shape the different phases within the research. Recognizing that we are not value-free, we consider it important to acknowledge and disclose our position. This transparency is crucial for understanding how various aspects of our identity may influence the various stages of the study. One of the researchers shares a similar cultural background with the participants. This fostered a safe environment where the fathers felt more comfortable and secure. The researcher’s deeper understanding of the father’s cultural and migration background, positively influenced the level of openness of the participants [67], and enabled the provision of a more reliable thick description and understanding of the Moroccan culture [68]. When there is a shared culture between the researcher and participants, it becomes easier to comprehend colloquial language and non-verbal communication cues. Having a shared background can be beneficial in research, but it can also lead to limitations. The researcher might make assumptions about shared experiences, resulting in biased ideas. In turn, participants may expect the interviewer to immediately understand them, making them less likely to elaborate on certain topics and hinder the collection of important information. Holmes even argues that attempting to adopt an insider or outsider position does not necessarily mean having that position [69]. Subsequently, it is questionable whether achieving such as position is even possible. Therefore, even if researchers and participants speak the same language and share the same background, it can still be challenging to communicate effectively.

With regard to language, in research, various considerations should be taken into account. The researchers spoke Dutch, English, and Moroccan Arabic, ensuring that Moroccan fathers could express themselves in the language they felt most comfortable with. However, this approach may have resulted in an underrepresentation of parents who do not speak any of these languages, such as Turkish. Although we conducted the interviews and focus group in the preferred languages of the participants, there are some challenges associated with being a multi-language researcher, such as conceptual similarities, timing of translation, the quality of a translator, and ethnocentricity [70, 71]. During one informal conversation with a Turkish father, the interview was conducted in Dutch and translated by a key figure who spoke Turkish. Using a non-professional translator might have resulted in the loss of data during translation [72]. With regard to the data collected in Moroccan Arabic, we checked the transcripts for accuracy in translation. We are aware that integrating the linguistic needs of participants and researchers can lead to practical and methodological challenges as the analysis was conducted on the translated version rather than the original data.

To ensure the risk and safety of both researchers and participants during the COVID-19 pandemic, data collection took place online. We are very much aware that conducting research with virtual technology does not simply replace in-person interviews. Conducting research online gave us less time to establish trust and rapport, and limited ability to observe body languages. Although we recognize that virtual qualitative research has its challenges, the benefits, especially during COVID-19 were greater [73].

## Conclusion

This study contributes to cross-cultural parenting research by including diverse perspectives and experiences from both the participants and the research team, fostering the development of more inclusive research and parenting programs. Specifically, our findings add to the existing literature by highlighting the needs and perspectives of fathers with a migration background. We recommend further studies that explore the contributions of fathers from various migration backgrounds in promoting their children’s healthy energy balance-related behaviors. Gaining deeper insights into their needs and perceptions can guide the development of better tailored and thereby more relevant and accessible parenting programs focused on parenting and healthy lifestyles. Both previous studies along with the results of this study demonstrate that children’s growing independence makes it increasingly challenging for parents to establish, monitor, and enforce rules regarding healthy behaviors. This underscores the importance of developing tailored age-specific communication strategies and tools that address the challenges associated with different developmental stages particularly during and after adolescence. In conclusion, parenting programs need to adopt a comprehensive approach, acknowledging multifaceted problems and prioritizing cultural and linguistic inclusivity. Fostering a good atmosphere through culturally responsive facilitation, inclusive language, mutual respect, and peer solidarity is essential for engaging fathers with a migration background in parenting programs. These principles should underpin the design of future parenting programs.

## Data Availability

This study analyzes qualitative data. The participants did not consent to have the full transcripts of the interviews and focus group made publicly available. Supporting passages from the data, such as quotes, are available within the text of this manuscript. The data supporting the findings of this study are not publicly available, since the data contains information that could compromise the privacy and consent of the participants, but are available from the principal researcher M.L.Overman (m.overman@maastrichtuniversity.nl) on reasonable request, and subset to approval from the Medical Ethical Committee of Amsterdam UMC, location VU Medical Centre.
